# Contribution of sex and gender roles to the incidence of post-infectious irritable bowel syndrome in a prospective study

**DOI:** 10.1038/s41598-023-45300-2

**Published:** 2023-11-09

**Authors:** Jae Ho Park, Sun Hyung Kang, Ju Seok Kim, Hee Seok Moon, Jae Kyu Sung, Hyun Yong Jeong

**Affiliations:** 1 Division of Gastroenterology, Department of Internal Medicine, Chungnam National University Sejong Hospital, Sejong, South Korea; 2 Chungnam National University School of Medicine, Daejeon, South Korea; 3 Division of Gastroenterology, Department of Internal Medicine, Chungnam National University Hospital, 282 Munhwa-ro, Jung-gu, Daejeon, 35015 South Korea

**Keywords:** Intestinal diseases, Human behaviour

## Abstract

Post-infectious irritable bowel syndrome (PI-IBS) occurs in about 10% of cases following gastroenteritis. The incidence of IBS is higher in females. However, it is not clear whether this is due to biological or psychosocial factors. We aimed to investigate the influence of gender roles on the incidence of PI-IBS, alongside traditional risk factors. Our study included 231 patients diagnosed with gastroenteritis who were hospitalized and treated with antibiotics between 2018 and 2021. The Korean Sex Role Inventory-Short Form (KSRI-SF), based on the Bem Sex Role Inventory (BSRI) was used to categorize patients (androgynous, masculine, feminine, and undifferentiated types). Six months after treatment, we conducted a telephone survey to confirm the presence of PI-IBS using the ROME IV criteria. Among the patients, 43.3% were female, and the mean age was 43.67 ± 16.09 years. After 6 months, 34 patients developed PI-IBS. Univariate analysis revealed that younger age, female sex, KSRI-SF undifferentiated type, and longer duration of antibiotic use independently influenced the occurrence of PI-IBS. Multivariate analysis showed that PI-IBS was associated with the KSRI-SF undifferentiated type and higher C-reactive protein (CRP) levels. Our study showed that the KSRI-SF undifferentiated type and high CRP levels at initial infection were associated with PI-IBS.

## Introduction

Female predominancy of irritable bowel syndrome (IBS) is well known and has been confirmed in recent studies^1,2^. Post-infectious IBS (PI-IBS), where infection is the clear causal factor, is also predominantly expressed in females^3^. Abdominal pain is an essential element for the diagnosis of IBS in the ROME IV criteria. Abdominal discomfort or pain were the diagnostic criteria in ROME III; however, abdominal discomfort was excluded from the diagnostic criteria in ROME IV, and the scope was expanded from relieving symptoms due to defecation to symptoms related to defecation. Therefore, the presence or absence of pain, a subjective complaint of the patient, has emerged as an important factor in the diagnosis of IBS, and the need to analyze the factors contributing to the subjective pain threshold has increased.

Sex-based differences in the prevalence of pain are well known^4,5^. Most population-based studies have found a higher prevalence of pain in females than in males, and it has been reported that females are more likely to express symptoms and respond to pain than males^6^. Abdominal pain is also more common in females, and several epidemiological studies have also revealed that abdominal pain is more common in IBS in females^7^. It is unclear whether this is due to biological sex or to the gender roles. A gender role is a role that is socially learned and considered desirable and has the characteristics of masculinity and femininity. According to previous studies on pain, it has been reported that people who identify with male gender roles are more tolerant of pain and people who identify with female gender roles have a lower pain threshold^8^. Additionally, in a previous study, it was reported that there was a difference in the rate of reporting the presence or absence of pain due to social gender roles^4^.

It has been suggested that IBS is influenced by the brain-gut axis^9,10^. Central modulation of the brain and peripheral pain reception from gut visceral hypersensitivity are known to interact and influence each other. There are several reports on anatomical and hormonal differences due to biological differences. However, there are few studies on the effects of social gender roles in the psychological domain. Therefore, we attempted to determine whether conventional factors, biological sex, and social gender roles (divided into androgynous, masculinity, femininity, and undifferentiated) affect abdominal pain complaints and, thus, the occurrence of PI-IBS.

## Results

### Patient characteristics

**Figure 1:**
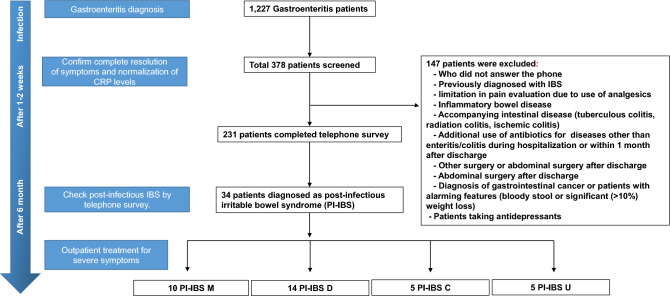
The study flow diagram. *D* Diarrhea type, *C* Constipation type, *CRP* C-reactive protein, *IBS* irritable bowel syndrome, *M* mixed type, *PI-IBS* post infectious irritable bowel syndrome, *U* undifferentiated type.

Of the 231 patients identified with acute gastroenteritis, 34 patients were diagnosed with PI-IBS (Fig. 1); the mean age of patients was 43.67 ± 16.09 years; 188 patients (81.4%) were < 60 years of age (Table 1). There were 109 (58.0%) males and 79 (42.0%) females under the age of 60 and 22 (51.2%) males and 21 (48.8%) females over 60 years of age. Differences in the incidence of abdominal pain and PI-IBS between men and women as well as mean age were observed, but were not associated with BMI, underlying diseases such as diabetes and blood pressure, smoking, or drinking. The KSRI-SF classifications were: androgynous type, 62 (26.8%); masculine type, 61 (26.4%); feminine type, 57 (24.7%); and undifferentiated type, 51 (22.1%) patients. In each group, 25 (40.32%), 21 (34.43%), 23 (40.35%), and 31 (60.8%) patients were females, respectively (Table 2).

**Table 1:** Baseline characteristics.

Patient-related characteristics							
Variable	ALL n = 231	No abdominal pain n = 177	Abdominal pain n = 54	*P*	No PI-IBS n = 197	PI-IBS n = 34	*p*
Age (years)	43.67 ± 16.09	44.98 ± 16.63	39.39 ± 13.41	0.013	44.61 ± 16.47	38.24 ± 12.5	0.012
< 60	188	139	49		157	31	
≥ 60	43	38	5		40	3	
Sex				0.008			0.044
Male	131	109	22		117	14	
Female	100	68	32		80	20	
BMI (kg/m^2^)	24.04 ± 3.51	23.80 ± 3.30	24.82 ± 4.05	0.06	23.93 ± 3.39	24.62 ± 4.12	0.363
< 23.0	80	66	14		70	10	
23.0–25.0	75	56	19		63	12	
≥ 25.0	76	55	21		64	12	
Underlying disease							
DM	37	30	7	0.484	33	4	0.464
HTN	21	17	4	0.623	18	3	0.953
Smoking				0.723			0.338
Yes	35	26	9		28	7	
No	196	151	45		169	27	
Drinking				0.704			0.454
Yes	82	64	18		68	14	
No	149	113	36		129	20

**Table 2:** Clinical finding of initial admission.

Variable	ALL n = 231	No abdominal pain n = 177	Abdominal pain n = 54	*P*	No PI-IBS n = 197	PI-IBS n = 34	*p*
Fever (Days)	0.86 ± 0.97	0.81 ± 0.99	1.04 ± 0.910	0.130	0.85 ± 0.99	0.91 ± 0.830	0.745
Fever							
Presence	151	111	40	0.125	128	23	0.091
Absence	80	66	14		69	11	
Diarrhea (n)	7.05 ± 5.90	6.74 ± 5.90	8.06 ± 5.88	0.152	6.96 ± 5.93	7.56 ± 5.80	0.585
Peak CRP (mg/dL)	10.56 ± 6.09	8.99 ± 5.19	15.7 ± 6.07	< 0.01	10.10 ± 6.13	13.22 ± 5.21	< 0.01
Antibiotics				0.803			0.090
Ciprofloxacin	155	119	36		134	21	
Ciprofloxacin + Metronidazole	39	28	11		32	7	
Ceftriaxone	28	23	5		24	4	
Ceftriaxone + Metronidazole	9	7	2		7	2	
Duration of antibiotic use (days)	10.79 ± 1.79	10.58 ± 1.69	11.48 ± 1.95	< 0.01	10.68 ± 1.74	11.41 ± 1.94	0.046
Gender role identity	Androgynous	52	10	0.002	54	8	0.030
	Masculine	50	11		53	8	
	Feminine	46	11		53	4	
	Undifferentiated	29	22		37	4	

*BMI* body mass index, *CRP* C-reactive protein, *DM* diabetes mellitus, *HTN* hypertension, *PI-IBS* post infectious irritable bowel syndrome.

### Characteristics of infection at initial admission

During hospitalization, fever occurred in 151 (65.4%) patients. The maximal number of diarrhea incidents per day was 7.05 ± 5.90 and peak C-reactive protein (CRP) was 10.56 ± 6.09 mg/dL. Four types of antibiotics were used in relation to the severity of the disease, causative organism, and patient's condition (ciprofloxacin, ciprofloxacin with metronidazole, ceftriaxone, and ceftriaxone with metronidazole); ciprofloxacin alone was the most common treatment (155 patients, 67.1%, Table 2). The average number of days of antibiotic use was 10.79 ± 1.79.

### Factors that affected the occurrence of PI-IBS

PI-IBS occurred in 34 patients (14.71%), 14 (41.2%) of whom were male and 20 of whom (58.8%) were female (*P* = 0.044) (Table 1). Most patients had IBS-diarrhea (n = 14), followed by those with IBS-mixed (n = 10), IBS-constipation (n = 5), IBS-unclassified (n = 5, Table 3). The PI-IBS group was younger (38.24 ± 12.50 vs. 44.61 ± 16.47 years) and had a longer period of antibiotics use (11.41 ± 1.94 vs. 10.64 ± 1.73 days) than the non-PI-IBS group. (Table 1, 2.)

**Table 3:** Clinical finding of Post infectious irritable bowel syndrome group.

Post infectious IBS	Total	n = 34	%	
PI-IBS type	Diarrhea	14	6.1	
	Constipation	5	2.2	
	Mixed	10	4.3	
	Undifferentiated	5	2.2	

	Abdominal pain		PI-IBS	
Factors	Univariate OR (95% CI)	P**	Univariate OR (95% CI)	P**
Sex				
Male	1.0 (Reference)		1.0 (Reference)	
Female	1.996 (1.154–3.454)	0.013	2.124 (1.009–4.468)	0.047
KSRI-SF				
Masculine type	1.0 (Reference)		1.0 (Reference)	
Feminine type	4.158 (1.909–9.053)	< 0.01	0.480 (0.136–1.699)	0.255

The effects of the clinical factors on the occurrence of PI-IBS were analyzed (Table 4). Univariate analysis revealed that younger age (odds ratio [OR] 0.971; 95% confidence interval [CI], 0.946–0.997), female sex (OR 2.124; 95% CI 1.009–4.468), the KSRI-SF undifferentiated type (OR 2.676; 95% CI 1.014–7.062), Peak CRP (OR 1.084; 95% CI 1.021–1.150) and longer duration of antibiotics use (OR 1.259; 95% CI 1.041–1.523) influenced the occurrence of PI-IBS.

**Table 4:** Risk factors of post-infectious irritable bowel syndrome.

Factors	Univariate OR (95% CI)	P**	Multivariate AOR (95% CI)	P**
Age	0.971(0.946–0.997)	0.030		
Sex (female)	2.124(1.009–4.468)	0.047		
BMI (Kg/m^2^)	1.072(0.964–1.192)	0.200		
DM	0.633(0.209–1.923)	0.420		
HTN	1.090(0.298–3.990)	0.896		
Smoking				
Never	1.0 (Reference)			
Present	1.508(0.597–3.806)	0.385		
Drinking				
Never	1.0 (Reference)			
Present	1.411(0.668–2.984)	0.367		
KSRI-SF				
Androgyny	1.0 (Reference)		1.0 (Reference)	
Masculinity	1.083(0.377–3.113)	0.882	1.381(0.464–4.110)	0.562
Femininity	0.520(0.147–1.836)	0.310	0.608(0.168–2.205)	0.449
Undifferentiated	2.676(1.014–7.062)	0.047	3.456(1.245–9.591)	0.017
Initial admission				
Fever duration (Days)	1.047(0.739–1.483)	0.796		
Diarrhea (Most frequent/day)	1.012(0.995–1.072)	0.687		
Antibiotics duration	1.259(1.041–1.523)	0.018	1.206(0.967–1.502)	0.096
Peak CRP	1.084(1.021–1.150)	0.008	1.070(1.002–1.143)	0.043
Procalcitonin	1.181(0.885–1.576)	0.259		
Antibiotics				
Ciprofloxacin	1.0 (Reference)			
Ciprofloxacin + Metronidazole	1.679(0.647–4.357)	0.286		
Ceftriaxone	1.188(0.371–3.807)	0.772		
Ceftriaxone + Metronidazole	2.079(0.393–10.994)	0.389		
Antibiotics				
One (Ciprofloxacin or ceftriaxone)	1.0 (Reference)			
Combined with metronidazole	1.710(0.729–4.009)	0.217		

*BMI* body mass index, *AOR* adjusted odds ratio, *CI* confidence interval, *DM* diabetes mellitus, *HTN* hypertension, *KSRI-SF* Korean Sex Role Inventory-Short Form.

In the multivariate analysis of these factors, the incidence of PI-IBS was higher in patients with higher peak CRP levels (OR, 1.070; 95% CI 1.002–1.143) and the KSRI-SF undifferentiated type (OR, 3.456; 95% CI 1.245–9.591).

### Effect of sex and gender roles on abdominal pain and PI-IBS

**Figure 2:**
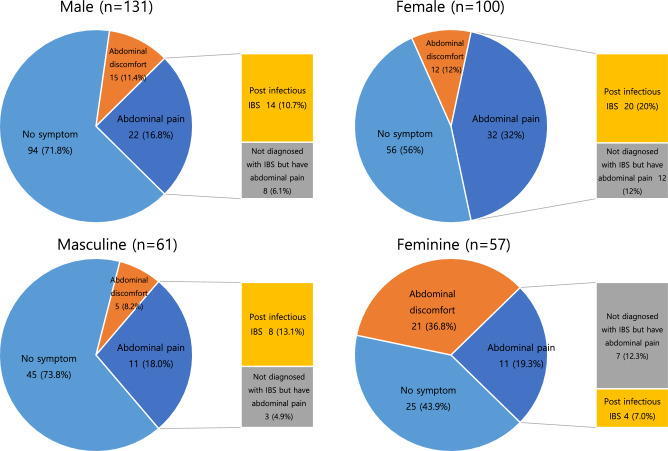
Incidence of abdominal pain and PI-IBS in patients with infectious colitis when stratified by sex (male, female) or gender (masculine, feminine). *IBS* irritable bowel syndrome, *PI-IBS* post infectious irritable bowel syndrome.

Abdominal pain and PI-IBS occurrence were analyzed by dividing the participants into biological sex (male and female) and the KSRI-SF masculine and feminine types (Table 5). Univariate analysis showed that the presence of abdominal pain was observed more commonly in females compared to males (OR 1.996, 95% CI 1.154–3.454, *P* = 0.013) and in the KSRI-SF feminine type compared to the masculine type (4.158, 95% CI 1.909–9.053, *P* < 0.01). In Fig. 2, we presented the severity of symptom expression categorized as asymptomatic, abdominal discomfort, and abdominal pain in male and female patients, as well as the occurrence rate of PI-IBS in patients with abdominal pain. It was observed that both the frequency of abdominal pain and the occurrence of PI-IBS were higher in females compared to males. When considering the KSRI-SF masculine type, those showing KSRI-feminine type reported a higher rate of abdominal discomfort and pain. In the case of PI-IBS, as shown in Table 3, female sex showed a significant association compared to male sex; but compared to the KSRI-SF masculine type, the feminine type showed no significant association (OR 0.480, 95% CI 0.136–1.699, *P* = 0.255).

**Table 5:** PI-IBS according to KSRI-SF gender role type.

Gender role identity by KSRI-SF^†^	n = 231 (%)	Sex			Abdominal pain n = 54	All	NRS^b^ (n = 54)		P**	PI-IBS^c^ n = 34
			N(%)	P**			No PI-IBS (n = 20)	PI-IBS (n = 34)		
Androgynous	62 (26.8)	Male	37(59.7)	0.663	4 (10.8%)	2.50 ± 1.29	2.50 ± 2.12	2.50 ± 1.51	1.00	4
		Female	25(40.3)		6 (24%)	2.83 ± 1.47				4
Masculine	61 (26.4)	Male	40(65.6)	0.199	6 (15.0%)	1.83 ± 0.75	2.33 ± 0.577	1.752 ± 0.707	0.23	5
		Female	21(34.4)		5 (23.8%)	2.80 ± 0.84				3
Feminine	57 (24.7)	Male	34(59.6)	0.570	5 (14.7%)	3.20 ± 0.83	3.43 ± 2.07	3.75 ± 0.957	0.780	2
		Female	23(40.4)		6 (26.1%)	4.33 ± 1.63				2
Undifferentiated	51 (22.1)	Male	20(39.2)	0.015	7 (35%)	4.57 ± 0.83	4.71 ± 1.113	5.64 ± 1.008	0.690	3
		Female	31(60.8)		15 (48.4%)	5.60 ± 0.99				11

*KSRI-SF*Korean Sex Role Inventory Short Form, *NRS* Numeric Rating Scale, *PI-IBS* post-infectious irritable bowel syndrome.

## Discussion

To our knowledge, this is the first study to analyze the effects of biological sex, gender roles, and various infectious factors on PI-IBS using the ROME IV criteria. In particular, the purpose of this study was to analyze the effects of biological and acquired gender roles on the occurrence of IBS. The analysis was conducted in selected groups, excluding those previously diagnosed with IBS and psychiatric illnesses or those receiving psychiatric medications. The incidence of PI-IBS has been reported to be approximately 20%, according to ROME III^11^, and 10–12.8%, according to ROME IV^3,12^. Our study, based on ROME IV, had an incidence of 14.7%. Our study reaffirmed previous findings that the incidence of PI-IBS increases with young age, severe enteritis, female sex, and psychological factors.

The ROME Foundation Working Team Report introduced PI-IBS as a complex and multifactorial disorder. Impaired visceral motility and visceral hypersensitivity, the microbiome, intestinal permeability, immune dysregulation^13–15^, the enteroendocrine pathway^16–18^, and genetics have been proposed as possible mechanisms of PI-IBS^3^. Younger age, female sex, psychological status, severity of initial enterocolitis, and pathogens (bacteria, viruses, protozoa, and parasites) have been reported as specific factors influencing the development of PI-IBS^3,12,19–22^. According to the ROME IV criteria, episodic abdominal pain is an essential symptom for the diagnosis of IBS^10^ and is the most significant symptom that leads to hospital visits. It also affects quality of life, the need for medical therapy, and social expenditure^23,24^. The presence or absence of pain has subjective characteristics, even when the same stimulation intensity is given. The threshold for determining the presence of pain involves complex factors resulting from the central and peripheral interactions^4^.

The biological differences between males and females are traditionally explained by chromosomes, gonads, hormones (estrogen and progesterone/testosterone), and external genitalia. Such differences cause anatomical differences and affect not only the spatial structure of the organs in the abdominal cavity but also the function of the gastrointestinal tract. There are also differences between males and females in the onset of IBS symptoms^7^. In one meta-analysis, females were more likely to report abdominal pain and constipation-related symptoms were predominant, while diarrhea-related symptoms were predominant in men^25^. Symptom differences according to menstruation, menopause, and hormone supplementation in women were analyzed, but insufficient data were reported.

In our study, females had more complaints of abdominal pain and there was a correlation with the occurrence of PI-IBS in the univariate analysis. In a previous study, when sigmoid stimulation was administered, discomfort was reported only in females with IBS, and not in males with or without IBS. This suggests that sex differences significantly influence perceptual sensitivity to rectosigmoid distension^26^. Numerous studies have focused on the role of estrogen as the cause of this difference^27,28^. In female patients with IBS, heightened neuronal activity in regions responsible for emotional processing, such as the insula, cingulate cortex, and amygdala, has been observed^29–31^. These differences are likely to contribute to visceral pain transmission in females.

Estrogen and progesterone have also been linked to intestinal permeability by increasing T cell and mast cell activities^32–34^. Reports indicate that 16–50% of patients with PI-IBS experience increased intestinal permeability^15,35^. Estrogen has been found to decrease permeability by increasing the tight junction protein^32^, while progesterone has been shown to reduce gut permeability by increasing occludin expression^36^. Hormonal ratios and concentrations influence intestinal permeability and thus IBS expression. In a study of prepubescent children, female patients with IBS showed an increased recovery of sucrose percentage and increased intestinal permeability compared to controls, while no difference was observed in males^37^. As such, the degree of hormonal effect on IBS may vary among the childhood, adulthood, and senior periods, suggesting that other factors may be more influential during each life stage.

Representative adverse psychological factors associated with IBS have been reported, including depression^18,20,38^, high stress and anxiety levels^39^, hypochondriasis, somatization, neuroticism, and adverse life events in the preceding three months. According to a meta-analysis, with the increasing number of studies investigating anxiety and IBS, anxiety and depression increase the odds of IBS approximately 3 times compared to controls^40^. In ROME IV, patients with severe pain, bloating, and somatization were higher than in ROME III. However, there was no difference in anxiety or depression^2^, suggesting that psychological factors contribute to ROME III or IV classifications.

Even if the same level of stimulation was given, the participants classified as masculine by the BSRI were more likely not to report pain than participants classified as feminine^4,8^. Whether socially-learned masculinity and femininity actually affect the occurrence of IBS is unclear. In this study, the authors hypothesized that social gender roles could influence the reporting of abdominal pain, which is indispensable for the diagnosis of IBS. According to the results of our study, the feminine gender role showed a correlation with abdominal pain but did not affect the occurrence of PI-IBS. The cause of these results may be a diagnostic feature of PI-IBS, which is chronic recurrent episodic abdominal pain related to defecation, stool form or frequency change that occurs after infection. The authors hypothesized that feminine gender role showed an association with abdominal pain, a component of the diagnosis of IBS, but the association with defecation- and stool- related was unclear and consequently did not show a significant association with PI-IBS. In addition, due to the nature of PI-IBS, it was estimated that factors such as infection and antibiotic use were dominant compared to other factors; therefore, the proportion of psychological causes decreased.

Patients classified as the BSRI undifferentiated type, which is typified by a low level of both masculine and feminine traits, exhibited a more negative self-concept compared to other BSRI types, and a correlation with depression is suggested^41–44^. The association of depression, anxiety, and somatization with IBS has been reported numerously^38,45^, and up to 60% of patients reported having major psychosocial problems^46^. An association with depression has also been reported in PI-IBS^47^. It has been reported that in patients diagnosed with depression, females are more likely to report pain than males^48^. In a study conducted in Brazil, the prevalence ratio of depression in the feminine gender role group was reported to be 2.2 times that of the masculine gender role group^49^. In another study of elderly population, the BSRI-undifferentiated type was reported to have an association with depression that was not present in the feminine type (BSRI-feminine type (OR 0.83 (0.64–1.07))/undifferentiated type (OR 1.22 (0.98–1.52))^44^. The authors considered the possibility of an association between KSRI-undifferentiated type and depression in the development of PI-IBS, given that the undifferentiated type has been shown to be associated with depression in a number of studies and exhibits depression-associated features such as a more negative self-concept compared with other BSRI types. Further validation is needed in future research. In the Korean nation-wide statistics published in 2022^50^, the lifetime prevalence of depressive disorder was reported to be 7.7%. The lifetime prevalence rates in males and females were 5.7 and 9.8%, respectively, with higher rates in females. Although our study excluded those who were diagnosed with depression and were taking medication to minimize psychological effects, psychological tests could not be performed on all participants.

Consistent with the above research results, the results of our study showed that the incidence of PI-IBS was increased in females, and the KSRI-SF feminine type was associated with increased abdominal pain, but did not increase the incidence of PI-IBS. It is possible that biological sex differences, including hormonal and other influences, alter bowel habits and increase the incidence of PI-IBS through an association with intestinal permeability and psychological factors. The association between the occurrence of PI-IBS and the KSRI-SF undifferentiated type suggests that psychological factors could also influence the occurrence of PI-IBS. The association of PI-IBS with the KSRI-SF undifferentiated type may be related to the high proportion of females with the KSRI-SF undifferentiated type, the relationship between the KSRI-SF undifferentiated type and depression, and the negative self-identity of patients with the KSRI-SF undifferentiated type itself. This needs to be elucidated in further research.

There are a few papers directly associating CRP levels with PI-IBS. Based on a small group study of 89 individuals following the ROME-III criteria, Wang et al. reported an association between high CRP levels and PI-IBS. In another study, although not statistically significant, it was reported that WBC count and CRP levels were higher in the IBS group at the onset of initial abdominal symptoms^51^. In our study, we found an association between a high peak CRP level and the occurrence of PI-IBS. Infectious gastroenteritis is considered the strongest risk factor for the development of PI-IBS. The authors speculate that a high level of CRP may contribute to the occurrence of PI-IBS by reflecting severe inflammation and potentially indicating mucosal damage. Further research is needed to confirm this.

This study had some limitations. Although we confirmed that there was no multicollinearity between the KSRI-undifferentiated type and sex, the proportion of females was higher in the KSRI-undifferentiated type. Considering the higher prevalence of depression in female and previous studies showing an association between reduced rectal mucosal blood flow and psychological morbidity in female with the BSRI-undifferentiated type, we cannot completely rule out the influence of sex. Given the multifactorial nature of PI-IBS, further research is needed to confirm this. In addition, although we excluded patients currently taking medication for depression, we did not perform a diagnostic test for depression on admission, so we cannot completely rule it out. Among the various factors that can affect IBS, visceral hypersensitivity and sex hormone concentrations/ratios could not be quantitatively measured. Owing to the nature of the questionnaire, it is possible that the results may have been influenced by the life cycle, education level regarding gender equality, education at the time of birth, occupation, and income, and it is difficult to control these factors. In addition, the participants’ diets were not controlled. Furthermore, the participants of this study were Koreans who are not representative of all Asian individuals.

In conclusion, associations between PI-IBS and female sex, KSRI-SF undifferentiated type, young age, and high CRP levels were observed. The KSRI-feminine type was associated with abdominal pain, but not PI-IBS, and it is important to carefully select and monitor patients with these risk factors to provide early diagnosis and treatment for PI-IBS.

## Methods

### Patients

Of the 1227 patients who were diagnosed with acute gastroenteritis, hospitalized, and treated with antibiotics at the Chungnam National University Hospital and Chungnam National University Sejong Hospital from January 1, 2018 to December 31, 2021, we prospectively enrolled those who consented to the study during hospitalization. A total of 378 patients were screened and 231 participants were finally enrolled. If the symptoms improved after antibiotic use, the patients were discharged from the hospital, and follow-ups were performed on an outpatient basis within 2 weeks to confirm that the inflammatory marker (c-reactive protein [CRP]) had improved, and the symptoms were recorded. After 6 months, an interview-administered questionnaire using a telephonic survey was conducted and the occurrence of PI-IBS was confirmed, based on the ROME IV criteria^9^. (Fig. 1).

The exclusion criteria were as follows: (i) patients who did not answer the phone; (ii) patients previously diagnosed with IBS; (iii) limitation in pain evaluation due to use of analgesics; (iv) inflammatory bowel disease (ulcerative colitis or Crohn’s disease); (v) accompanying intestinal disease (tuberculous colitis, radiation colitis, non-infectious diarrhea, ischemic colitis, eosinophilic Behçet’s disease, chronic diarrhea, or allergic colitis); (vi) additional use of antibiotics during hospitalization or within 1 month after discharge (pneumonia or urinary tract infection); (vii) other surgery or abdominal surgery after discharge; and (viii) diagnosis of gastrointestinal cancer or patients with alarming features (bloody stool or significant (> 10%) weight loss); (ix) Patients taking antidepressants. The ROME IV criteria were used for IBS diagnosis, and the Bristol stool form scale was used to classify IBS subtypes into IBS-diarrhea, IBS-constipation, IBS-mixed, and IBS-undifferentiated.

This study was reviewed and approved by the Institutional Review Board of the Chungnam National University Hospital Institutional Review Board (IRB file No. CNUH 2018-04-011) and was performed in accordance with the ethical standards set by the 1964 Declaration of Helsinki and its later amendments or comparable ethical standards. Informed consent was obtained from all patients.

### Defining gender roles

The Bem Sex Role Inventory (BSRI) is the most commonly used and validated measure of gender roles^52^. Bem classifies gender roles as masculine and feminine, androgyny (high score for both masculine and feminine traits), and undifferentiated (low score for both masculine and feminine traits). In this study, the short form of the Korean Sex Role Inventory (KSRI-SF), based on the BSRI, was used in consideration of Korean culture and language^52,53^. The short form of the BSRI is relatively simple and easy to use, and it shows similar results to the BSRI in classifying masculinity and femininity^52,54,55^. The questionnaire consists of 10 items, and each item is scored on a 7-point scale. There are 5 items for masculinity and 5 items for femininity. As suggested by the authors, gender role identities were classified using the median-split method.

### Statistical analysis

Data were classified as means ± standard deviation for continuous variables and percentages for categorical variables. For the baseline characteristics, clinical data, and group comparison, categorical variables were analyzed by χ2 tests and continuous variables by t-tests. Univariate and multivariate factor analyses were performed using binomial logistic regression. In the multivariate analysis, a backward binomial logistic regression test was performed using all variables with a *P*-value of 0.1 or less in the univariate analysis. *P*-values < 0.05 were considered statistically significant. Statistical analyses were performed using the SPSS software version 26 for Windows (SPSS Inc., Chicago, IL, USA).

## Data Availability

The datasets generated and analyzed in this study are not publicly available for privacy reasons but are available from the corresponding author upon reasonable request.
